# Efficacy and safety of photothermal biomodulated platelet-rich plasma versus standard platelet-rich plasma for facial rejuvenation: a prospective split-face randomized study

**DOI:** 10.1038/s41598-026-54310-9

**Published:** 2026-06-01

**Authors:** Rungsima Wanitphakdeedecha, Ma. Patricia Gertrude Camille Rojas Ollero, Woramate Bhorntarakcharoen, Sariya Sittiwanaruk, Wilailuck Phokai, Thanakorn Woramongkol, Thanya Techapichetvanich, Pitchaya Maneeprasopchoke, Panwadee Thongjaroensirikul, Ya-Nin Nokdhes, Pornsuk Yamlexnoi, Onjira Meethong

**Affiliations:** 1 Department of Dermatology, Faculty of Medicine Siriraj Hospital, Mahidol University, Bangkok, Thailand; 2 Department of Dermatology, Fatima University Medical Center, Antipolo, Philippines

**Keywords:** Facial rejuvenation, Photothermal biomodulation, Platelet-rich plasma, Preconditioning, Wrinkles, Diseases, Health care, Medical research

## Abstract

Autologous platelet-rich plasma (PRP) regenerative properties are used to treat clinical manifestations of skin aging. This study assessed the efficacy and safety of photothermal biomodulated PRP (PTBM-PRP) compared to standard PRP for skin rejuvenation. Prospective, split-face, randomized study including Thai volunteers (30–60 years) with Fitzpatrick skin type III-IV and moderate severity photoaging or higher conducted at Siriraj Hospital (Bangkok, Thailand). Face sides were randomized to receive intradermal autologous PTBM-PRP or PRP injections in three sessions at one-month intervals, with follow-up visits at one, three, and six months. PRP was preconditioned using the MCT System (Meta Cell Technology, Sant Cugat del Vallès, Spain) preset with the Exosomes program (467 nm continuous light [1 J/cm^2^], 37 °C, 10 min). We evaluated changes in skin surface characteristics, pigmentation, redness, and biomechanical properties (i.e., firmness, elasticity, and viscoelasticity), perceived facial improvement, and perceived pain. We included 28 volunteers (96.4% women) with a mean (SD) age of 40.82 (8.95) years and mostly skin type IV (67.86%). PTBM-PRP decreased fine lines maximum depth (*p* = 0.041) and indentation index (*p* = 0.005), folds maximum depth (*p* = 0.032) and indentation index (*p* < 0.001), and wrinkles maximum depth (*p* = 0.024) and indentation index (*p* = 0.041) in the periorbital area. PRP transiently decreased folds indentation index (*p* = 0.008) and wrinkles maximum depth (*p* = 0.0499) (PTBM-PRP vs. PRP wrinkles maximum depth *p* = 0.038 and indentation *p* = 0.018). Improvements in pigmentation, redness, and biomechanical properties were similar for both treatments. Patients perceived both treatment effects mostly as good or excellent improvement, with slightly favorable pain scores for PTBM-PRP vs. PRP, particularly at visit 3 (*p* = 0.006). Compared to standard PRP, PTBM-preconditioned PRP for facial skin rejuvenation results in greater improvements in fine lines, folds, and wrinkles parameters, with sustained effects for more than six months, supporting a PTBM protocol use to enhance the regenerative capacity of PRP in skin rejuvenation.

**Supplementary Information:**

The online version contains supplementary material available at 10.1038/s41598-026-54310-9.

## Background

Skin aging results from the combined effects of chronological aging and extrinsic or environmental factors, primarily UV radiation from the sun (i.e., photoaging), leading to cumulative changes in skin structure, function, and appearance^1^. Alterations in the extracellular matrix of the dermis, including reduced collagen density and thickness, and changes in elastic fibers, result in loss of skin stiffness and elasticity, which manifest as wrinkling and sagging^1,2^. UV and blue light-induced photoaging further contribute to the appearance of more pronounced and numerous wrinkles, and additionally, altered pigmentation. Moreover, skin aging is associated with decreased sebum content, hydration, and altered vascularization^1,2^. Skin aging is more apparent in the face and forearms, which are more exposed to sunlight photodamaging effects.

Numerous approaches to treat clinical manifestations of skin aging and restore skin structure and function have emerged, such as injectable compounds containing active substances and dermal fillers^3,4^. Among these minimally invasive approaches, dermal injections with autologous platelet-rich plasma (PRP) are used to promote dermal reconstruction through the regenerative action of growth factors and other molecular mediators secreted by platelets^5,6^. Injection of PRP triggers platelet activation by tissue contact, leading to the release growth factors and cytokines^7^. PRP is obtained from patient’s blood following a simple, cheap procedure, and its application is therefore feasible in many settings. Moreover, unlike other popular procedures such as laser treatments, PRP treatment is suitable for all skin phototypes^7^. Given its autologous nature, it is generally well tolerated and has few contraindications^7^. The regenerative properties of PRP have been explored to treat multiple conditions across different areas^6^. In dermatology, PRP has been shown to improve skin conditions and has shown optimal outcomes in skin rejuvenation and cosmetology applications, resulting in improved appearance, texture, skin tone, and reduced lines, wrinkles, and pores^8–10^.

The platelet-derived molecules that mediate the PRP effects are released either free or encapsulated in exosomes^11^. These extracellular vesicles contain bioactive molecules, such as growth factors, cytokines, chemokines, and RNAs (mRNA and microRNA), and have been proposed as the main mediators of PRP regenerative effects^12,13^. Exosomes have shown anti-aging properties, and are widely used in dermatology and cosmetic applications^14,15^. Of the multiple strategies investigated to modulate exosome biogenesis and release, such as physical stimuli, pharmacological or other external agents, as well as physiological and environmental factors, photobiomodulation with blue light in the 455–480 nm range has been shown to enhance the properties of exosomes secreted from human umbilical cord mesenchymal stem cells^16^. Moreover, photothermal biomodulation (PTBM), consisting of simultaneous controlled exposure to light and temperature, has been used for PRP preconditioning to enhance its properties^17^. Photothermal biomodulated PRP (PTBM-PRP) has shown promising results to treat diabetic ulcers as well as in facial and hands skin rejuvenation procedures, but evidence is still scarce^18–21^.

While the regenerative capacity of PRP on dermal tissues and its clinical effects on skin rejuvenation have been investigated, studies assessing the efficacy of PTBM-PRP for skin rejuvenation are limited. This prospective, split-face, randomized study aimed to evaluate the efficacy and safety of PTBM-PRP compared to PRP on facial rejuvenation.

## Methods

### Study design and setting

This was a prospective, split-face, randomized, single-center study including healthy male and female volunteers between 30 and 60 years with moderate severity photoaging or higher according to the Glogau classification to compare the efficacy and safety of preconditioned PRP by photothermal biomodulation (PTBM-PRP) vs. standard PRP injections for skin rejuvenation. The study was conducted between October 2024 and October 2025 at Siriraj Skin Laser Center, Faculty of Medicine Siriraj Hospital, Mahidol University, Thailand. The treatment was administered in three sessions at one-month intervals (visits 1 to 3) and participants were followed-up at 1, 3, and 6 months after the end of treatment (visits 4 to 6); therefore, the study included 6 visits (denoted as V1, V2, V3, V4, V5, and V6, following a chronological order). Figure S1 includes a diagram of the study design.

This study was conducted in accordance with the principles of the Helsinki Declaration (Fortaleza, Brazil) and later amendments. All patients provided written informed consent to participate in the study and for publication of their data. The study protocol was approved by the Siriraj Institutional Review Board, Faculty of Medicine Siriraj Hospital, Mahidol University, Thailand (COA no. Si561/2024) and was registered with the Thai Clinical Trials Registry (TCTR; www.thaiclinicaltrials.org) with the identification code TCTR20251020002. The trial record was first submitted on 10/10/2025 and first posted on 20/10/2025. As the first participant was enrolled on 11/10/2024, the trial was retrospectively registered.

### Study patients

This study included Thai individuals of Asian descent with Fitzpatrick skin type III–V. They all agreed to participate in the study voluntarily and to receive the study treatment at the frequencies and locations specified in the protocol.

Pregnant or lactating volunteers were excluded. Volunteers with anemia, coagulation or platelet disorders, serious chronic or psychiatric diseases, or who were immunocompromised, as well as those taking medications that could affect blood clotting, such as aspirin or anticoagulants, were also excluded. Additional exclusion criteria were previous cosmetic procedures, including filler, botulinum toxin, or PRP injections, thread lifts, laser, or other energy-based device treatments within the past 6 months. Volunteers with dermatitis, wounds, or active skin infections in the treatment area, as well as those with a history of keloid scarring were also excluded. Volunteers regularly using nonsteroidal anti-inflammatory drugs (NSAIDs), as well as those requiring hormonal contraception (oral, intramuscular, or implantable), were excluded. Finally, individuals who did not consent to be photographed for evaluation of treatment outcomes were not eligible to participate.

### Intervention

#### Blood sample collection, processing, and PRP preconditioning protocol

In each treatment session (V1 to V3), 20 mL of blood were drawn from each volunteer via cephalic or basilic veins using a syringe prefilled with 2 mL of acid citrate dextrose solution A (ACD-A). The mixture was transferred to Minos^®^ PRP kits (Medistar, Seoul, Korea) and centrifuged for 5 min at 3500 rpm. The resulting PRP fraction (3–4 mL) was recovered and split equally for preconditioning by photothermal biomodulation (PTBM) using the MCT System (Meta Cell Technology, Sant Cugat del Vallès, Spain) or for direct injection.

The MCT System, consisting of the MCT kit (Meta Cell Technology, Sant Cugat del Vallès, Spain) and the MCT unit (Meta Cell Technology, Sant Cugat del Vallès, Spain), is a novel PTBM device with controlled wavelength and temperature settings for preconditioning PRP. Half of the recovered PRP (1.5–2 mL) was inserted into the MCT kit, a 6 × 12 cm medical device made of a synthetic polymer with optimal optical properties to ensure that most of the emitted light reaches the sample and designed to maximize the laser interface^22^. The PRP sample was subject to PTBM using the Exosomes program, an automatic preset program that emits continuous light of 467 nm at a 1 J/cm^2^ intensity and at a stable temperature of 37 °C during 10 min, to generate preconditioned autologous PRP containing platelets primed for exosome release (PTBM-PRP).

#### Injection procedures

Before treatment, facial skin was cleaned using a mild, fragrance-free, non-comedogenic cleanser and water. An anesthetic lidocaine-prilocaine cream (EMLA^®^ cream, AstraZeneca, Wilmington, DE, USA) was applied and both sides of the face were covered with plastic for 45 min, and then the anesthetic cream was washed off. Each side of the face received the assigned treatment, PTBM-PRP or PRP, through intradermal injections using a 30G needle spaced 1 cm. The total volume injected ranged 0.5–1 mL in the cheek area, 0.25–0.5 mL in the periorbital area, and 0.25–0.5 mL in the nasolabial area (Fig. S2).

### Randomization and blinding

Randomization of facial sides was performed using a computer-generated random sequence to assign each side of the face to receive either PTBM-PRP or standard PRP. The randomization sequence was generated by an independent staff member not involved in treatment administration using an online randomization program (www.randomization.com). Allocation concealment was ensured using sealed, opaque envelopes prepared by an independent staff member. The treatment syringes were masked before administration to maintain blinding of the injector, participants, and outcome assessors. The injector, participants, and investigators involved in follow-up assessments and data analysis were all blinded to treatment allocation throughout the study.

### Efficacy outcomes, variables and assessments

The primary outcome of this study was changes at V2, V3, V4, V5, and V6 with respect to baseline (V1) in parameters related to skin surface properties, including texture (i.e., roughness), fine lines, wrinkles, and folds, and parameters related to biomechanical properties (skin distensibility/firmness, elasticity, and viscoelasticity) measured with a Cutometer. Secondary efficacy outcomes included changes in additional parameters related to skin surface properties, including pores and volume depression, pigmentation, vascular status, as well as subjective patients’ perceptions of facial skin improvement throughout study visits (Table S1). Objective efficacy assessments were performed at six study visits: visit 1 (V1) (at baseline, before the first treatment session), V2 and V3, before the second and third treatment sessions, and during three follow-up visits 1, 3, and 6 months after the end of treatment (V4, V5, and V6, respectively). Subjective assessments were performed at all visits except V1 (Fig. S1).

Skin surface properties were skin texture and wrinkles and were assessed using the Antera 3D Diagnostic System^®^ (Miravex Limited, Ireland) on three areas: periorbital, cheek, and nasolabial fold areas. This system provides multispectral, three-dimensional imaging for quantitative assessment of skin topography and chromophore content. The parameters analyzed to evaluate changes in surface microrelief and in wrinkle morphology were those related to skin texture, including texture roughness (Ra), mean pore volume (mm^3^), and mean pore area (mm^2^), fine lines, wrinkles, and folds parameters, including maximum depth (mm) and indentation index (a.u.), and volume depression (mm^3^). The Antera 3D System^®^ was also used to evaluate changes in pigmentation, measured as relative melanin concentration, and vascularity, measured as the relative hemoglobin concentration.

Biomechanical properties were skin distensibility/firmness, elasticity, and viscoelasticity, and were evaluated using a Cutometer^®^ MPA-580 probe (Courage + Khazaka Electronic GmbH, Köln, Germany) on the cheek area at the intersection of an imaginary vertical line drawn from the lateral canthus and an imaginary horizontal line drawn from the alar base using a 2-mm probe aperture under a constant negative pressure of 450 mbar. The cutometer parameters R0, R2, R5, R6, and R7 were recorded to quantify skin pliability/firmness (R0, mm); elasticity, including net elasticity (R5, %) and the elastic portion (R7, %); and viscoelasticity, including overall viscoelasticity (R2, %) and viscoelastic portion (R6, %). Decreased R0 and R6 values indicate increased skin firmness and reduced viscoelasticity, whereas increased R2, R5, and R7 values indicate increased skin elasticity and viscoelastic recovery. These parameters provide a clinically meaningful measurement of skin biomechanical properties.

Moreover, photographs were obtained by standard photographic imaging of the face at 5 angles (67.5°, 45°, 0°, − 45°, − 67.5°), corresponding to two images per hemiface and one frontal image, using the OMNIA^®^ Imaging System (Canfield Scientific Inc., NJ, USA) at each study visit. The OMNIA^®^ Imaging System is a standardized facial imaging platform that provides controlled lighting, fixed subject positioning, and multi-angle high-resolution image acquisition to ensure reproducible photographs across study visits. Subjective efficacy measures were based on the patient’s blinded self-evaluation of each face side on a quartile scale: <0%, deterioration; 0%, no improvement; 1–25%, slight improvement; 26–50%, moderate improvement; 51–75%, good improvement; 76–100%, excellent improvement; no Investigator’s/Physician’s Global Assessment (IGA) was performed.

### Safety outcomes and assessments

Safety (secondary objective) was evaluated by recording adverse events occurring after each treatment session and throughout the study period, including bruising, erythema, edema, irritation/dermatitis, post-inflammatory hyperpigmentation, hypopigmentation, oozing, crusting, infection, and scarring. Moreover, patients evaluated pain immediately after each treatment session on a visual analog scale (VAS), ranging from 0, no pain to 10, the most painful in life.

### Statistical analysis

A sample size of 28 volunteers was considered sufficient to assess the study outcomes. Sample size was calculated using McNemar’s test of equality of paired proportions for a split-face design. The assumed effect size was informed by a prior study of PTBM-PRP (620 nm) reporting 40–60% physician-perceived improvement versus standard PRP^19^. As no published data are available for 467 nm photothermal biomodulation of PRP, a conservative minimum paired-proportion difference of 0.40 was assumed, with a discordant-pair proportion of 0.60. With a two-sided α = 0.05 and 80% power, the required sample size was 26 participants, as determined using nQuery Advisor, (Statistical Solutions, Saugus, MA, USA), which was increased to 28 participants to allow for 5% potential missing data.

Qualitative variables were summarized as frequencies and percentages, and quantitative variables as means and standard deviations (SD). Longitudinal changes in study variables over time and between treatments were analyzed using a mixed-effects model with restricted maximum likelihood estimation (REML), to account for repeated measurements within subjects in the split-face design. The Bonferroni multiple-comparison test was applied to assess differences from baseline (V1) and between treatments at each visit. Pain scores were compared between treatments using a paired t-test. Statistical significance was set at a two-sided α < 0.05. All statistical analyses were performed using IBM SPSS Statistics version 29.0 (IBM Corp., Armonk, NY, USA) and GraphPad Prism version 10.6 (GraphPad Software Inc., San Diego, CA, USA).

## Results

### Demographic characteristics of study volunteers

The study included 28 volunteers, mostly women (*n* = 27, 96.4%), with a mean (SD) age of 40.82 (8.95) years. Fitzpatrick skin type was IV in 19 patients (67.86%), III in 8 volunteers (28.57%), and V in 1 patient (3.57%). All study volunteers completed the three treatment sessions (V1 to V3) and attended V4, 26 attended V5, and 27 volunteers attended V6. A flow diagram of study patients is included in Fig. S3.

### Effects on skin surface characteristics

**Fig. 1:**
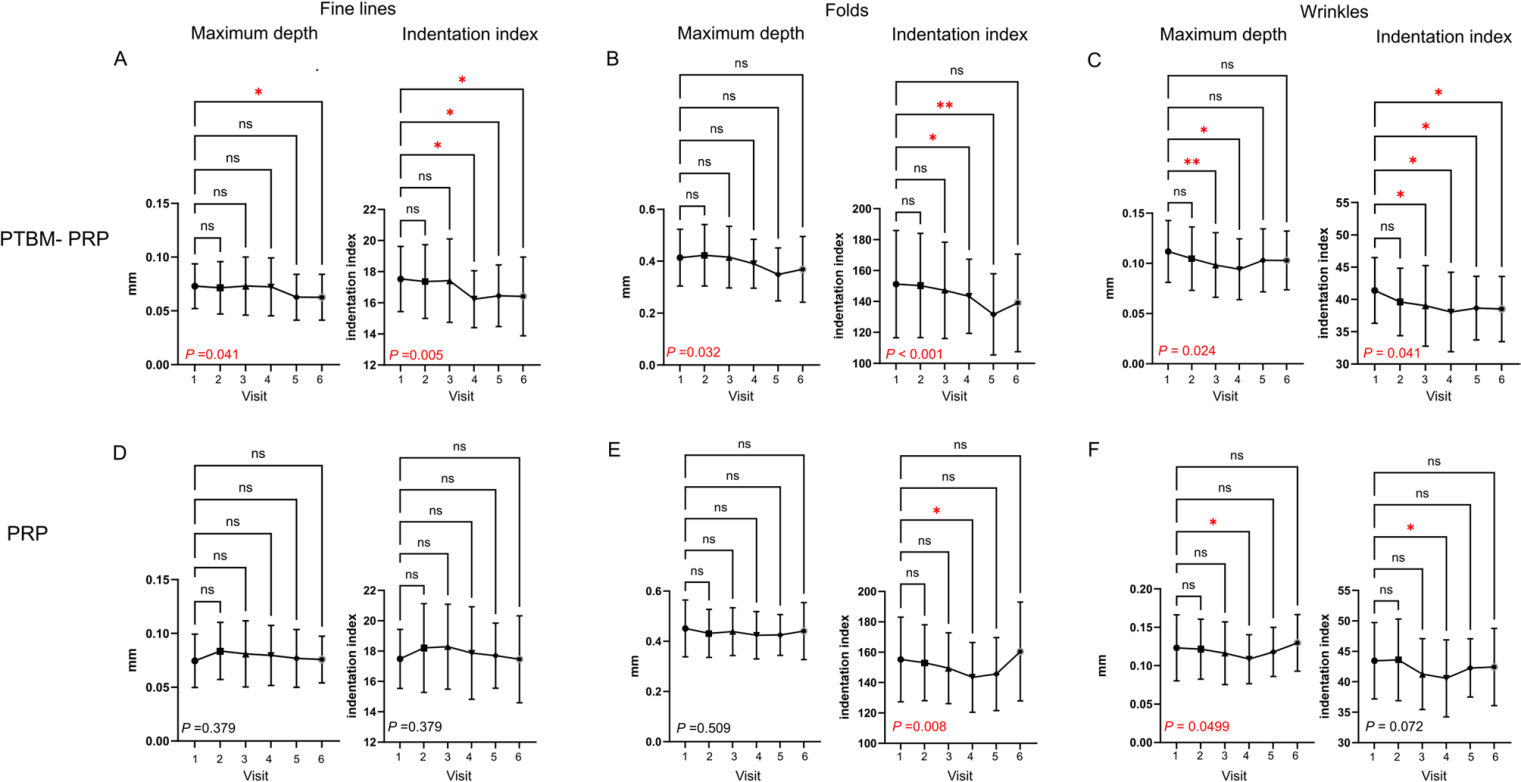
Assessment of fine lines, folds, and wrinkles in the periorbital area. Changes in maximum depth (mm) and indentation index of fine lines (**A**, **D**), folds (**B**, **E**), and wrinkles (**C**, **F**) in the periorbital area measured using the Antera 3D System, throughout study visits in facial sides treated with photothermal biomodulated platelet-rich plasma (PTBM-PRP) (**A**–**C**) and platelet-rich plasma (PRP) (**D**–**F**). Data points represent the mean and error bars represent the standard deviation. Bonferroni’s multiple comparison *p < 0.05 and **p < 0.01 for changes at each study visit compared to V1 (baseline). The *p*-value for the time effect analyzed using a mixed-effects model with restricted maximum likelihood estimation (REML) is shown inside each graph. NLF, nasolabial fold; ns, non-significant.

Of all the parameters assessing skin surface characteristics, the maximum depth and indentation index of fine lines, folds, and wrinkles significantly decreased in the periorbital area on the PTBM-PRP treated side, but not on the standard PRP treated side (Fig. 1, Table S2). These changes were significant after the end of treatment (V4, V5, or V6), except for the maximum depth of folds. Overall effects of PTBM-PRP treatment over time were statistically significant for all variables: fine lines (maximum depth, *p* = 0.041 and indentation index *p* = 0.005), folds (maximum depth, *p* = 0.032 and indentation index *p* < 0.001), and wrinkles (maximum depth, *p* = 0.024 and indentation index, *p* = 0.041). Conversely, overall effects of PRP treatment were statistically significant for two variables, folds indentation index (*p* = 0.008) and wrinkles maximum depth (*p* = 0.0499), due to a transient significant decrease at V4 (Fig. 1, Table S2).

**Fig. 2:**
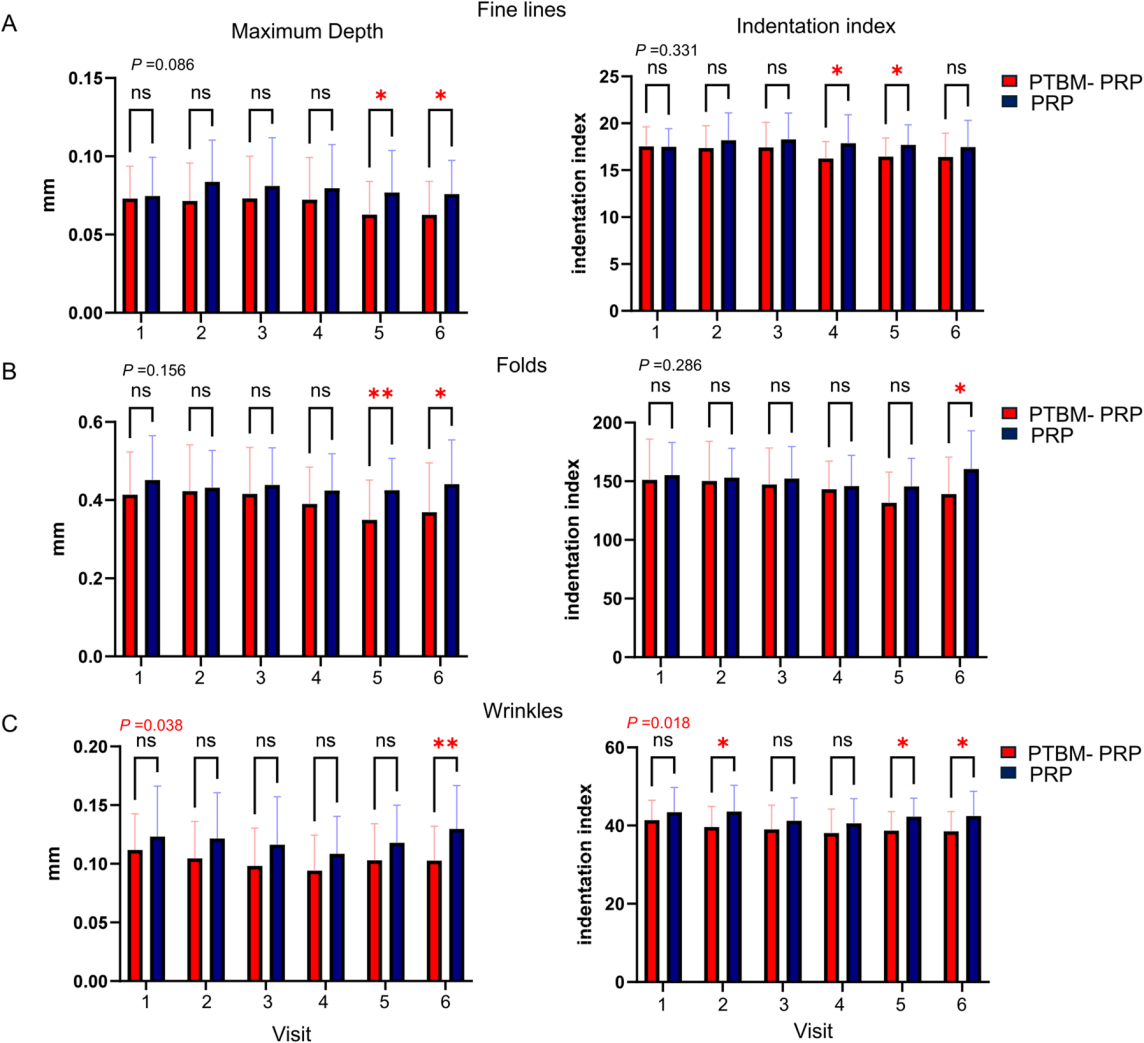
Comparison of fine lines, folds, and wrinkles between treatments. Comparison of mean maximum depth and indentation index of fine lines (**A**), folds (**B**), and wrinkles (**C**) between photothermal biomodulated platelet-rich plasma (PTBM-PRP) (red) and platelet-rich plasma (PRP) (blue) treated facial sides throughout study visits in periorbital areas. Error bars represent the standard deviation. Bonferroni’s multiple comparison *p < 0.05 and **p < 0.01 for differences between treatments at each study visit. The *p*-value for the treatment effect analyzed using a mixed-effects model with restricted maximum likelihood estimation (REML) is shown above each graph. ns, non-significant.

Comparison between treatments showed significantly decreased fine lines, folds, and wrinkles parameters on the PTBM-PRP-treated vs. the PRP-treated side, particularly during follow-up visits. These differences yielded significant treatment effects for wrinkles maximum depth (*p* = 0.038) and indentation (*p* = 0.018) (Fig. 2 and Table S2).

The remaining skin surface variables analyzed (i.e., roughness, mean pore volume, and area, and volume depression) showed no significant changes in the periorbital area throughout visits, with no differences between treatment sides (Figure S4). Likewise, skin surface parameters remained unchanged in the cheek and nasolabial fold areas throughout study visits, with no differences between treatment sides (Figs. S5–S8).

#### Effects on pigmentation and vascular status

**Fig. 3:**
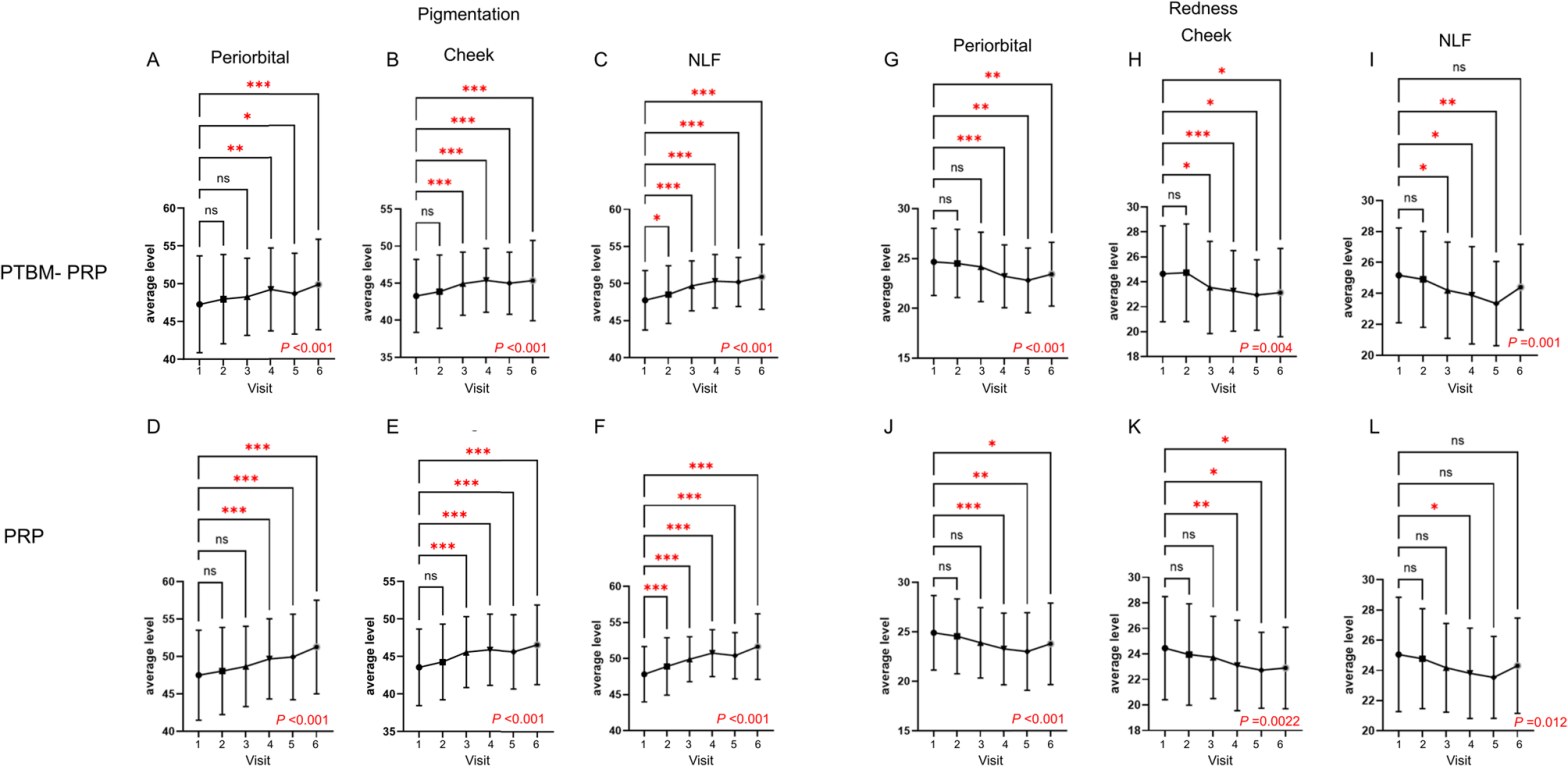
Pigmentation and redness measured with the Antera 3D System throughout study visits. Changes in mean pigmentation (**A**–**F**) and redness (**G**–**L**) measured using the Antera 3D throughout study visits in facial sides treated with photothermal biomodulated platelet-rich plasma (PTBM-PRP) (**A**–**C** and **G**–**I**) and platelet-rich plasma (PRP) (**D**–**F** and **J**–**L**). Error bars represent the standard deviation. Bonferroni’s multiple comparison *p < 0.05, **p < 0.01, and ***p < 0.001 for changes at each study visit compared to V1 (baseline). The *p*-value for the time effect analyzed using a mixed-effects model with restricted maximum likelihood estimation (REML) is shown inside each graph. NLF, nasolabial fold; ns, non-significant.

Pigmentation levels measured using the Antera 3D System^®^ showed a gradual increase from BL across all facial areas on both treatment sides, with significant changes observed at follow-up visits (V4, V5, and V6) in the periorbital area, at V3 and follow-up visits in the cheek area, and at all visits in the nasolabial fold area. A significant time effect was observed for all areas and both treatments (mixed-effects model, *p* < 0.001) (Fig. 3). Regarding redness, mean levels gradually decreased from BL across all facial areas on both treatment sides, with *p*-values reaching significance at different timepoints depending on the area. A significant time effect was observed for all areas and both treatments (mixed-effects model, *p* < 0.001) (Fig. 3). The two treatments, PTBM-PRP and PRP, showed similar effects on pigmentation and redness at all timepoints (mixed-effect model *p*, ns for the three areas) (Fig. S9).

#### Effects on skin biomechanical properties: firmness, elasticity, and viscoelasticity

**Fig. 4:**
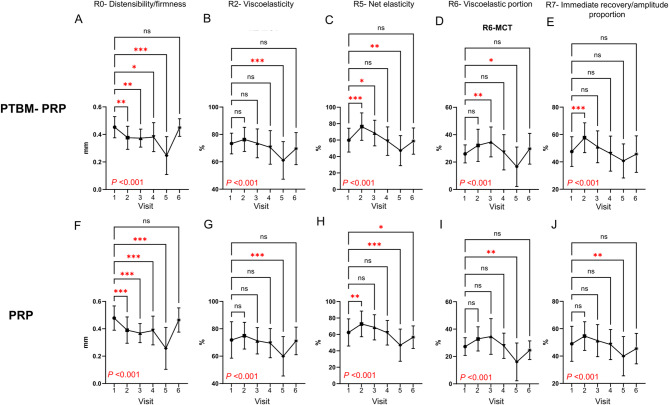
Cutometer distensibility/firmness, elasticity, and viscoelasticity measures throughout study visits. Changes in mean R0 (**A**, **F**), R2 (**B**, **G**), R5 (**C**, **H**), R6 (**D**, **I**), and R7 (**E**, **J**) biomechanical properties throughout study visits in facial sides treated with photothermal biomodulated platelet-rich plasma (PTBM-PRP) (A-E) and platelet-rich plasma (PRP) (F-J). Error bars represent the standard deviation. Bonferroni’s multiple comparison *p < 0.05, **p < 0.01, and ***p < 0.001 for changes at each study visit compared to V1 (baseline). The *p*-value for the time effect analyzed using a mixed-effects model with restricted maximum likelihood estimation (REML) is shown inside each graph. ns, non-significant.

R0 values were significantly lower at V2, V3, V4, and V5 compared to V1 (baseline), but not at V6, on both treatment sides, indicating improved firmness (Fig. 4). Elasticity and viscoelasticity parameters (R2, R5, R6, and R7) transiently increased shortly after treatment, but overall changes varied and were largely modest across visits and treatments (Fig. 4). Both PTBM-PRP and PRP injections resulted in significant effects on all biomechanical parameters after treatment (mixed effects model *p* < 0.001 across parameters and treatments).

The effects of PTBM-PRP vs. PRP treatment on biomechanical properties were similar across study visits, with no significant treatment effects observed (mixed-effect model *p*, ns for both treatments) (Figure S10).

### Patient-perceived improvement

**Fig. 5:**
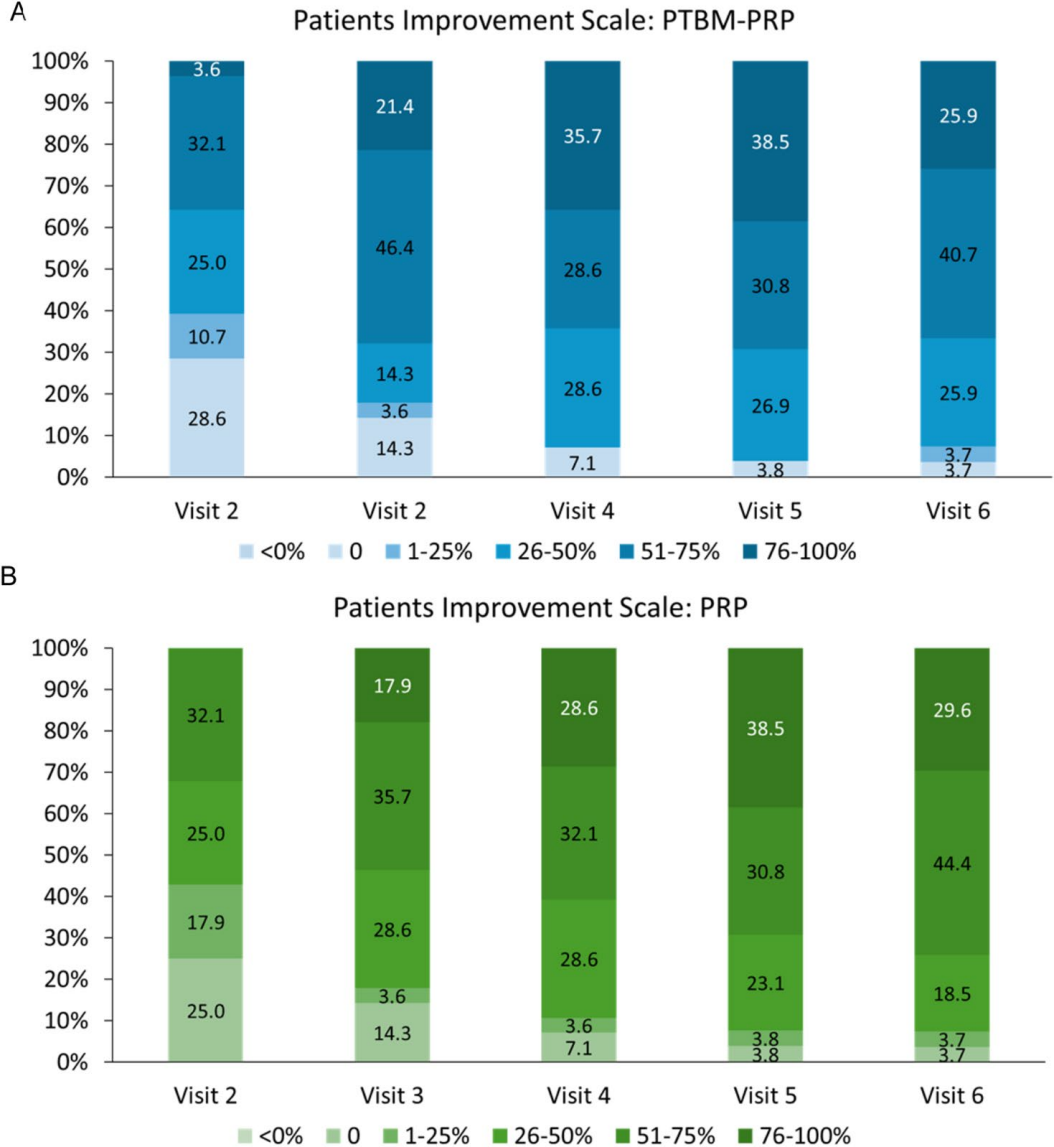
Perceived patient’s improvement. Patients’ improvement scale throughout study visits in facial sides treated with photothermal biomodulated platelet-rich plasma (PTBM-PRP) (**A**) and platelet-rich plasma (PRP) (**B**). Bars represent the percentages of the indicated responses throughout study visits.

Most patients (> 50%) perceived the treatment effects as a good or excellent improvement from V2 (before the second treatment injection) on both treatment sides. Overall, 69.3% of patients perceived good/excellent improvement after 3 months of PTBM-PRP and PRP treatment, and only a small proportion of patients (< 10.7%) perceived slight or no improvement at follow-up visits for both treatments (Fig. 5).

### Safety outcomes

Pain scores showed minimal fluctuations throughout study visits, with minor differences between facial sides (Table 1). In this study, two mild, self-limited local reactions were reported (each 1/28; 3.6%). One participant developed bilateral post-injection bruising after the first treatment that resolved spontaneously within 3 days, and one participant reported mild bilateral injection-site pruritus after the third treatment that resolved spontaneously within 24 h. No patients discontinued the intervention due to adverse events.

**Table 1:** Pain scores across treatment sessions and treatment sides.

Treatment session	Photothermal biomodulated PRP		PRP		*P*-value
	Mean (SD)	Range	Mean (SD)	Range	
1st treatment	4.07 (1.92)	1–9	4.21 (1.91)	1–8	0.547
2nd treatment	5.04 (2.17)	1–9	5.11 (2.15)	1–9	0.573
3rd treatment	3.64 (1.99)	0–7	3.89 (2.08)	0–8	0.006
Overall	4.25 (2.09)	0–9	4.40 (2.09)	0–9	0.096

PRP, platelet-rich plasma; SD, standard deviation.

## Discussion

This prospective, split-face, randomized study comparing the efficacy of PTBM-PRP and PRP on skin rejuvenation parameters showed that PTBM-PRP resulted in improved facial skin rejuvenation outcomes compared with standard PRP. Specifically, PTBM-PRP, but not standard PRP, improved fine lines, folds, and wrinkles parameters in the periorbital area, with effects persisting for 6 months after the end of treatment, indicating durable treatment effects. Both PTBM-PRP and PRP resulted in comparable improvements in biomechanical properties, including distensibility/firmness, elasticity and viscoelasticity, as well as pigmentation and redness, based on Antera 3D System^®^ measurements. Overall, treatment effects were perceived as good or excellent, with only a small number of mild and self-limited adverse effects reported and similar pain scores for the two treatments.

In this study using a split-face design, PTBM-PRP effectively improved skin surface properties in the periorbital area, measured as decreased values for parameters associated with fine lines, folds, and wrinkles, also known as crow’s feet. Remarkably, maximum depth and indentation index progressively decreased, even after the end of treatment, and the improvement persisted during at least six months. The effect size of PTBM-PRP was modest and restricted to the periorbital area, likely because aging manifestations are often more pronounced and appear earlier in this area than in the cheeks and nasolabial folds regions^23^. Unlike PTBM-PRP, PRP showed minimal, transient, and largely non-significant improvements in folds and wrinkles parameters, which did not persist beyond one-month post-treatment.

Although the effects of PTBM-PRP on objective wrinkle measures have not been previously assessed, several studies have evaluated PRP for wrinkle improvement^24–26^. However, study design and methods were highly heterogeneous, with high variability regarding changes in skin surface assessments precluding direct comparisons among studies. Moreover, PRP preparation and administration protocols are diverse, further precluding firm conclusions. Consequently, despite the reported general benefits of PRP on skin aging manifestations, some authors argue that robust, high-quality, consistent data regarding PRP effects on skin surface parameters is still limited^24,26^. Nevertheless, PRP is widely recognized to reduce facial wrinkle counts and volume^25^. Using a split-face design and objective measures, this study provides robust evidence that PRP preconditioning by PTBM improves fine lines, folds, and wrinkles in the periorbital area.

To our knowledge, this is the first study designed to assess the effects of PTBM on the regenerative capacity of PRP, by comparing PTBM-PRP and standard PRP effects on facial skin. Previous studies have shown that PTBM-PRP improves facial skin laxity and resulted in high patients’ and physicians’ satisfaction for facial skin rejuvenation^19,20^. Similar to our study, a pilot study comparing PTBM-PRP and standard PRP effects on hands skin rejuvenation reported a trend towards improved outcomes on hands receiving PTBM-PRP, supporting our results^21^. Nevertheless, given the scarce evidence of PTBM-PRP in this setting, our results provide valuable information on the efficacy and safety of PRP preconditioning to enhance its regenerative properties.

Our results showing improved distensibility/firmness, elasticity, and viscoelasticity after both treatments align with existing literature, demonstrating robust and convincing effects of PRP on these parameters^9,10,25^. The observed increase in pigmentation is most likely explained by hemosiderin deposition due to injection-related microvascular injury, rather than increased melanin production or inadequate photoprotection. However, unlike previous studies, the PRP-based treatments assessed in our study lacked effects in other parameters^9^. These differences may be explained by the heterogenous methods among studies, including protocols and assessments. Nevertheless, the use of objective measures provides compelling evidence for the benefits of PRP-based treatments on biomechanical properties. In this regard, our study indicates that PRP preconditioning boosts its regenerative properties, reflected by its unique effects on several skin surface parameters.

Our study assessed a PTBM protocol to precondition platelets to enhance exosome release. This study focused on clinical outcomes and therefore, the effects of PTBM on exosome and growth factors release remained unaddressed. In this regard, the protocol used in this study has been shown to achieve a mean (SD) exosome yield of 2.5 (1.4) × 10^11^ particles/mL from PRP^27^. In line with these observations, PTBM has been shown to increase growth factor content in preconditioned PRP preparations using protocols designed to enhance growth factor release^17^. Therefore, the improved outcomes of PTBM-PRP vs. PRP likely reflect increased exosome release from preconditioned platelets.

Results from this study should be interpreted in the context of minor limitations associated with the parameters analyzed, which were limited to clinical measures. While our study used numerous objective robust measures, changes in skin structure associated with PRP injections remained unaddressed. Nevertheless, the study focused on all clinical manifestations of skin aging and was therefore a comprehensive analysis. Moreover, the study protocol was retrospectively registered in the Thai Clinical Trials Registry. Although the inclusion criteria considered females and males, only male was included in the study, limiting the external validity of this study’s findings to the female population. Despite these limitations, this study used a split-face and randomized design and was therefore robust. Unlike other studies with a placebo-controlled design, PRP was used as comparator, with demonstrated effects on skin rejuvenation, and therefore, the study was highly rigorous. Moreover, outcome measures were mostly objective, providing conclusive evidence regarding the improved efficacy of PTBM-PRP compared to standard PRP on facial skin rejuvenation. PRP is widely used in diverse regenerative medicine applications, including dentistry, maxillofacial surgery, orthopedics and sports medicine, as well as gynecology and reproductive medicine, among others^6^. The demonstrated benefits of PTBM-mediated PRP preconditioning in skin regeneration to reverse the clinical manifestations of skin aging suggest that this strategy could be translated to other clinical settings. By enhancing the regenerative capacity of PRP, PTBM may open new avenues for more effective regenerative therapies and broaden the therapeutic potential of PRP-based approaches.

## Conclusions

Compared to standard PRP, PTBM-preconditioned PRP for facial skin rejuvenation results in greater improvements in fine lines, folds, and wrinkles parameters, with effects persisting for more than six months after treatment. Other skin characteristics, including biomechanical properties, pigmentation and vascular status, showed comparable improvements between PTBM-PRP and PRP. Overall, these results support the use of a PTBM protocol to enhance the regenerative capacity of PRP in skin rejuvenation and may open new avenues for its application in other clinical areas.

## Supplementary Information

Below is the link to the electronic supplementary material.

Supplementary Material 1

## Data Availability

The datasets generated during and/or analyzed during the current study are availablefrom the corresponding author on reasonable request.

## Declarations

### Medical writing, editorial, and other assistance

Medical writing assistance was provided by Meta Cell Technology S.L.
