# Supplementary material for: Efficacy and safety of photothermal biomodulated platelet-rich plasma versus standard platelet-rich plasma for facial rejuvenation: a prospective split-face randomized study
Source: Sci Rep. 2026 Jun 1;16:25017. doi: 10.1038/s41598-026-54310-9 (PMC13462578; doi:10.1038/s41598-026-54310-9)
Supplement: Supplementary file 1 [file 41598_2026_54310_MOESM1_ESM.docx]

# Supplementary Materials for the Submission “Efficacy and Safety of Photothermal Biomodulated Platelet-Rich Plasma Versus Standard Platelet-Rich Plasma for Facial Rejuvenation: A Prospective Split-Face Randomized Study” by Wanitphakdeedecha *et al*.

**Authors:**

Rungsima Wanitphakdeedecha^1^ (ORCID 0000-0002-3926-2193),

Ma. Patricia Gertrude Camille Rojas Ollero^1, 2^ (ORCID 0009-0003-0467-6454),

Woramate Bhorntarakcharoen^1^ (ORCID 0009-0002-1092-455X),

Sariya Sittiwanaruk^1^ (ORCID 0009-0005-5236-4193),

Wilailuck Phokai^1^ (ORCID 0009-0000-7446-2038),

Thanakorn Woramongkol^1^ (ORCID 0009-0009-0148-3490),

Thanya Techapichetvanich^1^ (ORCID 0000-0001-9590-8887),

Pitchaya Maneeprasopchoke^1^,

Panwadee Thongjaroensirikul^1^ (ORCID 0009-0003-5663-4790),

Ya-Nin Nokdhes^1^ (ORCID 0000-0001-5844-6038),

Pornsuk Yamlexnoi^1^,

Onjira Meethong^1^

**Affiliations:**

^1^Department of Dermatology, Faculty of Medicine Siriraj Hospital, Mahidol University. Bangkok, Thailand.

^2^Department of Dermatology, Fatima University Medical Center. Philippines.

**Corresponding author:**

Rungsima Wanitphakdeedecha, M.D., M.A., M.Sc., Ph.D. (ORCID 0000-0002-3926-2193)

Email: rungsima.wan@mahidol.ac.th

# Supplementary Tables

| **Table S1.** Study efficacy outcomes, assessments and variables. | | |  |  |
| --- | --- | --- | --- | --- |
| **Outcomes** | | **Outcome measures/variables** | **Units** | **Equipment** |
| **Objective efficacy outcomes (V1 to V6)** | |  |  |  |
| Surface characteristics | Texture | Roughness | Ra | Antera 3D® System |
|  | Fine lines | Maximum depth | mm | Antera 3D® System |
|  |  | Indentation index | a.u. | Antera 3D® System |
|  | Folds | Maximum depth | mm | Antera 3D® System |
|  |  | Indentation index | a.u. | Antera 3D® System |
|  | Wrinkles | Maximum depth | mm | Antera 3D® System |
|  |  | Indentation index | a.u. | Antera 3D® System |
|  | Pores | Mean pore volume | mm^3^ | Antera 3D® System |
|  |  | Mean pore area | mm^2^ | Antera 3D® System |
|  | Total concave volume of surface irregularities | Volume depression | mm^3^ | Antera 3D® System |
| Pigmentation | Melanin | Relative melanin concentration | a.u. | Antera 3D® System |
| Vascular status | Erythema/vascularity | Relative hemoglobin concentration | a.u | Antera 3D® System |
| Biomechanical properties | Distensibility/firmness | R0 | mm | Cutometer® |
|  | Elasticity | R5- net elasticity | % | Cutometer® |
|  |  | R7- elastic portion | % | Cutometer® |
|  | Viscoelasticity | R2- overall viscoelasticity | % | Cutometer® |
|  |  | R6- viscoelastic portion | % | Cutometer® |
| **Subjective efficacy outcomes (V2 to V6)** | |  |  |  |
| Perceived efficacy | Patient’s perceived efficacy | Treatment perceived results | Quartile scores | Evaluation of clinical photographs |
| a.u., arbitrary units; V, visit. | | | | |

| **Table S2.** Analysis of changes in maximum depth and indentation index of fine lines, folds, and wrinkles in the periorbital area across study visits and treatments. | | | | | | |
| --- | --- | --- | --- | --- | --- | --- |
|  | **Visit 1 (BL)** | **Visit 2** | **Visit 3** | **Visit 4** | **Visit 5** | **Visit 6** |
| **Fine lines maximum depth (mm)** |  |  |  |  |  |  |
| PTBM-PRP, *mean (SD)* | 0.0729 (0.0208) | 0.0714 (0.0243) | 0.0731 (0.0270) | 0.0723 (0.0269) | 0.0627 (0.0213) | 0.0626 (0.0213) |
| P-value vs. BL | N/A | >0.99 | >0.99 | >0.99 | 0.295 | **0.027** |
| P-value time effect | **0.041** | | | | | |
| PRP, *mean (SD)* | 0.0746 (0.0247) | 0.0837 (0.0266) | 0.0811 (0.0308) | 0.0796 (0.0279) | 0.0768 (0.0269) | 0.0758 (0.0217) |
| P-value vs. BL | N/A | 0.251 | 0.679 | >0.99 | >0.99 | >0.99 |
| P-value time effect | 0.379 | | | | | |
| P-value PTBM-PRP vs. PRP | 0.781 | 0.081 | 0.305 | 0.323 | **0.045** | **0.029** |
| P-value treatment effect | 0.086 | | | | | |
| **Fine lines indentation index (a.u.)** |  |  |  |  |  |  |
| PTBM-PRP, *mean (SD)* | 17.53 (2.09) | 17.37 (2.37) | 17.42 (2.68) | 16.23 (1.82) | 16.45 (1.98) | 16.41 (2.53) |
| P-value vs. BL |  | >0.99 | >0.99 | **0.043** | **0.014** | **0.036** |
| P-value time effect | **0.005** | | | | | |
| PRP, *mean (SD)* | 17.49 (1.95) | 18.2 (2.92) | 18.29 (2.80) | 17.87 (3.05) | 17.69 (2.14) | 17.46 (2.86) |
| P-value vs. BL | N/A | 0.242 | 0.203 | >0.99 | >0.99 | >0.99 |
| P-value time effect | 0.200 | | | | | |
| P-value PTBM-PRP vs. PRP | 0.933 | 0.254 | 0.243 | **0.021** | **0.040** | 0.161 |
| P-value treatment effect | 0.101 | | | | | |
| **Folds maximum depth (mm)** |  |  |  |  |  |  |
| PTBM-PRP, *mean (SD)* | 0.4137 (0.1096) | 0.4227 (0.1185) | 0.4159 (0.1188) | 0.3903 (0.0940) | 0.3492 (0.1021) | 0.3688 (0.1267) |
| P-value vs. BL | N/A | >0.99 | >0.99 | >0.99 | 0.090 | 0.940 |
| P-value time effect | **0.032** | | | | | |
| PRP, mean (SD) | 0.4513 (0.1137) | 0.4317 (0.0957) | 0.4386 (0.0952) | 0.4243 (0.0943) | 0.4252 (0.0813) | 0.4407 (0.1136) |
| P-value vs. BL | N/A | 0.890 | >0.99 | 0.652 | 0.724 | >0.99 |
| P-value time effect | 0.509 | | | | | |
| P-value PTBM-PRP vs. PRP | 0.214 | 0.760 | 0.432 | 0.188 | **0.007** | **0.040** |
| P-value treatment effect | 0.156 | | | | | |
| **Folds indentation index (a.u.)** |  |  |  |  |  |  |
| PTBM-PRP, *mean (SD)* | 151.20 (34.64) | 150.30 (33.69) | 147.20 (31.17) | 143.30 (23.98) | 131.60 (26.22) | 139.10 (31.54) |
| P-value vs. BL | N/A | >0.99 | 0.920 | **0.037** | **0.002** | 0.080 |
| P-value time effect | **<0.001** | | | | | |
| PRP, *mean (SD)* | 155.20 (27.87) | 153.00 (25.01) | 149.50 (23.35) | 143.40 (22.96) | 145.60 (24.07) | 160.50 (32.58) |
| P-value vs. BL | N/A | >0.99 | >0.99 | **0.022** | 0.270 | >0.99 |
| P-value time effect | **0.008** | | | | | |
| P-value PTBM-PRP vs. PRP | 0.636 | 0.737 | 0.519 | 0.703 | 0.062 | **0.019** |
| P-value treatment effect | 0.286 | | | | | |
| **Wrinkles maximum depth (mm)** |  |  |  |  |  |  |
| PTBM-PRP, *mean (SD)* | 0.1118 (0.0307) | 0.1046 (0.0315) | 0.0983 (0.0322) | 0.0941 (0.0303) | 0.1029 (0.0314) | 0.1028 (0.0293) |
| P-value vs. BL | N/A | 0.624 | **0.007** | **0.012** | 0.203 | 0.250 |
| P-value time effect | **0.024** | | | | | |
| PRP, *mean (SD)* | 0.1233 (0.0430) | 0.1215 (0.0391) | 0.1162 (0.0408) | 0.1085 (0.0319) | 0.1180 (0.0320) | 0.1297 (0.0368) |
| P-value vs. BL | N/A | >0.99 | 0.831 | **0.013** | >0.99 | >0.99 |
| P-value time effect | **0.0499** | | | | | |
| P-value PTBM-PRP vs. PRP | 0.254 | 0.087 | 0.074 | 0.092 | 0.106 | **0.005** |
| P-value treatment effect | **0.038** | | | | | |
| **Wrinkles indentation index (a.u.)** |  |  |  |  |  |  |
| PTBM-PRP, *mean (SD)* | 41.39 (5.10) | 39.62 (5.23) | 39.01 (6.24) | 38.06 (6.14) | 38.66 (4.92) | 38.50 (5.05) |
| P-value vs. BL |  | 0.501 | **0.024** | **0.027** | **0.013** | **0.018** |
| P-value time effect | **0.041** | | | | | |
| PRP, *mean (SD)* | 43.45 (6.27) | 43.59 (6.71) | 41.25 (5.82) | 40.57 (6.32) | 42.27 (4.78) | 42.43 (6.34) |
| P-value vs. BL | N/A | >0.99 | 0.693 | **0.021** | >0.99 | >0.99 |
| P-value time effect | 0.072 | | | | | |
| P-value PTBM-PRP vs. PRP | 0.184 | **0.020** | 0.178 | 0.146 | **0.017** | **0.023** |
| P-value treatment effect | **0.018** | | | | | |
| BL, baseline; PRP, platelet-rich plasma; PTBM-PRP, photothermal biomodulated platelet-rich plasma; SD, standard deviation; V, visit. | | | | | | |

# Supplementary Figures

**Figure S1. Study design diagram including treatment and follow-up visits and assessments.**

**Figure S2. Injection protocol.** Photothermal biomodulated platelet-rich plasma (PTBM-PRP) and platelet-rich plasma (PRP) injections in different facial areas.

**Figure S3.** CONSORT flow diagram of patient enrollment, allocation, follow-up, and analysis.

**Figure S4. Assessment of roughness, mean pore volume, mean pore area, and volume depression in the periorbital area.** Changes in roughness (A,E), mean pore volume (B,F), mean pore area (C,G), and volume depression (D,H) measured using the Antera 3D System, throughout study visits in facial sides treated with photothermal biomodulated platelet-rich plasma (PTBM-PRP) (A-D) and platelet-rich plasma (PRP) (E-H), and comparisons between facial sides receiving PTBM-PRP (red) and PRP (blue) injections (I-L). Error bars represent the standard deviation. Bonferroni’s multiple comparison for changes at each study visit compared to V1 (baseline) and between treatments yielded non-significant *p*-values. The *p*-value for the time and treatment effects analyzed using a mixed-effects model with restricted maximum likelihood estimation (REML) was non-significant. ns, non-significant.

**Figure S5. Assessment of fine lines, folds, and wrinkles in the cheeks.** Changes in mean maximum depth and indentation index of fine lines (A,D), folds (B,E), and wrinkles (C,F) in the cheeks measured using the Antera 3D System, throughout study visits in facial sides treated with photothermal biomodulated platelet-rich plasma (PTBM-PRP) (A-C) and platelet-rich plasma (PRP) (D-F). Error bars represent the standard deviation. Bonferroni’s multiple comparison for changes at each study visit compared to V1 (baseline) and between treatments yielded non-significant *p*-values. The *p*-value for the time effect analyzed using a mixed-effects model with restricted maximum likelihood estimation (REML) was non-significant. ns, non-significant.

**Figure S6. Assessment of roughness, mean pore volume, mean pore area, and volume depression in the cheeks.** Changes in roughness (A,E), mean pore volume (B,F), mean pore area (C,G), and volume depression (D,H) measured using the Antera 3D System, throughout study visits in facial sides treated with photothermal biomodulated platelet-rich plasma (PTBM-PRP) (A-D) and platelet-rich plasma (PRP) (E-H). Error bars represent the standard deviation. Bonferroni’s multiple comparison for changes at each study visit compared to V1 (baseline) and between treatments yielded non-significant *p*-values. The *p*-value for the time and treatment effects analyzed using a mixed-effects model with restricted maximum likelihood estimation (REML) was non-significant. ns, non-significant.

**Figure S7. Assessment of fine lines, folds, and wrinkles in the nasolabial folds (NLF).** Changes in mean maximum depth and indentation index of fine lines (A,D), folds (B,E), and wrinkles (C,F) in the nasolabial folds using the Antera 3D System, throughout study visits in facial sides treated with photothermal biomodulated platelet-rich plasma (PTBM-PRP) (A-C) and platelet-rich plasma (PRP) (D-F). Error bars represent the standard deviation. Bonferroni’s multiple comparison for changes at each study visit compared to V1 (baseline) and between treatments yielded non-significant *p*-values. The *p*-value for the time effect analyzed using a mixed-effects model with restricted maximum likelihood estimation (REML) was non-significant. ns, non-significant.

**Figure S8. Assessment of roughness, mean pore volume, mean pore area, and volume depression in the nasolabial folds (NLF).** Changes in roughness (A,E), mean pore volume (B,F), mean pore area (C,G), and volume depression (D,H) measured using the Antera 3D System, throughout study visits in facial sides treated with photothermal biomodulated platelet-rich plasma (PTBM-PRP) (A-D) and platelet-rich plasma (PRP) (E-H). Error bars represent the standard deviation. Bonferroni’s multiple comparison for changes at each study visit compared to V1 (baseline) and between treatments yielded non-significant *p*-values. The *p*-value for the time and treatment effects analyzed using a mixed-effects model with restricted maximum likelihood estimation (REML) was non-significant. ns, non-significant.

**Figure S9. Comparison of pigmentation and redness measured with the Antera 3D System between treatments.** Comparison of mean pigmentation (A-C) and redness (D-F) between photothermal biomodulated platelet-rich plasma (PTBM-PRP) (red) and platelet-rich plasma (PRP) (blue) treated facial sides throughout study in periorbital (A,D), cheek (B,E), and NLF areas (C,F). Error bars represent the standard deviation. Bonferroni’s multiple comparison was used to assess differences between treatments. The *p*-value for the treatment effect analyzed using a mixed-effects model with restricted maximum likelihood estimation (REML) was non-significant for all measurements. NLF, nasolabial fold; ns, non-significant.

**Figure S10. Comparison of distensibility, elasticity, and viscoelasticity measures between treatments.** Comparison of mean R0 (A), R2 (B), R5 (C), R6 (D), and R7 (E) cutometer measurements between photothermal biomodulated platelet-rich plasma (PTBM-PRP) (red) and platelet-rich plasma (PRP) (blue) treated facial sides throughout study visits. Error bars represent the standard deviation. Bonferroni’s multiple comparison was used to assess differences between treatments. The *p*-value for the treatment effect analyzed using a mixed-effects model with restricted maximum likelihood estimation (REML) was non-significant for all measurements. ns, non-significant.
